# Monthly Record of Deaths in the City of Buffalo

**Published:** 1858-08

**Authors:** H. D. Garvin

**Affiliations:** Health Physician


ART. X.—*Report of Mortality in Buffalo for the Month of June*, 1858. By H. D. GARVIN, M. D., Health Physician.

| DISEASES. | No. | Males. | Females. | No Sex given. |
|---|---|---|---|---|
| ACCIDENTAL, | | | | |
| Burning, | 2 | 1 | 1 | |
| Drowning, | 10 | 9 | 1 | |
| Albuminuria, | 1 | | 1 | |
| Apoplexy, | 2 | | 2 | |
| Atrophia, | 1 | 1 | | |
| Bronchitis, | 3 | 2 | 1 | |
| Cancer of Breast, | 1 | | 1 | |
| Child Birth, | 1 | | 1 | |
| Cholera Infantum, | 1 | 1 | | |
| Convulsions, | 13 | 7 | 5 | |
| Congestion, | 1 | 1 | | |
| Congestion of Liver, | 1 | | 1 | |
| Dropsy, | 2 | 1 | 1 | |
| Dysentery, | 1 | | 1 | |
| Enteritis, | 1 | 1 | | |
| Epilepsy, | 1 | | 1 | |
| Fever, Typhoid, | 4 | 2 | 1 | |
| " Scarlet, | 5 | 2 | 3 | |
| " Puerperal, | 1 | | 1 | |

### REGISTER OF MORTALITY—Continued.

| DISEASES. | No. | Males. | Females. | No Sex given. |
|---|---|---|---|---|
| Heart Disease, | 2 | 1 | 1 | |
| Hæmorrhage, | 2 | | 2 | |
| Hydrocephalus, | 6 | 3 | 3 | |
| Inanition, | 2 | 1 | 1 | |
| Insanity, | 1 | | 1 | |
| Marasmus, | 3 | 2 | 1 | |
| Meningitis, | 1 | 1 | | |
| Old Age, | 3 | 2 | 1 | |
| Paralysis, | 3 | 1 | 2 | |
| Phthisis, | 23 | 14 | 9 | |
| Pleurisy, | 1 | | 1 | |
| Pneumonia, | 2 | 1 | 1 | |
| Pulmonary Apoplexy, | 1 | | 1 | |
| Puerperal Convulsions, | 1 | | 1 | |
| Peritonitis, | 1 | | 1 | |
| Still Born, | 6 | 4 | 2 | |
| Sun Stroke, | 2 | 2 | | |
| Syphilitic-Tertiary, | 1 | | 1 | |
| Tabes Mesenterica, | 1 | 1 | | |
| Unknown, | 1 | 1 | | |
| Total, | 126 | | | |

### SEXES.

| | |
|---|---|
| Males, | 71 |
| Females, | 55 |
| Sex not given, | 0 |
| Total, | 126 |

### AGES.

| | | | |
|---|---|---|---|
| Still-born, | 6 | Between 20 years and 30 years, | 14 |
| 1 day, | 2 | " 30 " " 40 " | 12 |
| 1 day and 30 days, | 6 | " 40 " " 50 " | 11 |
| Between 1 month and 6 months, | 15 | " 50 " " 60 " | 8 |
| " 6 months and 12 " | 6 | " 60 " " 70 " | 1 |
| " 1 year " 3 years, | 17 | " 70 " " 80 " | 3 |
| " 3 " " 5 " | 3 | " 80 " " 90 " | 3 |
| " 5 " " 10 " | 6 | " 90 " " 100 " | 0 |
| " 10 " " 20 " | 7 | " 100 " | 0 |
| | 68 | | 22 |
| | | | 126 |

Ages not given, 6

Total, 126

### NATIVITIES.

| | | | |
|---|---|---|---|
| American, | 78 | Prussian, | 0 |
| German, | 19 | Italian, | 0 |
| Irish, | 20 | French, | 2 |
| English, | 2 | Scotch, | 2 |
| Canadian, | 0 | Isle of Man, | 1 |
| Holland, | 0 | Country not given, | 2 |

Total, 126

